# Fundamental mechanisms of telomerase action in yeasts and mammals: understanding telomeres and telomerase in cancer cells

**DOI:** 10.1098/rsob.160338

**Published:** 2017-03-22

**Authors:** Christine A. Armstrong, Kazunori Tomita

**Affiliations:** Chromosome Maintenance Group, UCL Cancer Institute, University College London, 72 Huntley Street, London WC1E 6DD, UK

**Keywords:** shelterin, telomere length homeostasis, replicative senescence, Hayflick limit, t-stumps, *S. pombe*

## Abstract

Aberrant activation of telomerase occurs in 85–90% of all cancers and underpins the ability of cancer cells to bypass their proliferative limit, rendering them immortal. The activity of telomerase is tightly controlled at multiple levels, from transcriptional regulation of the telomerase components to holoenzyme biogenesis and recruitment to the telomere, and finally activation and processivity. However, studies using cancer cell lines and other model systems have begun to reveal features of telomeres and telomerase that are unique to cancer. This review summarizes our current knowledge on the mechanisms of telomerase recruitment and activation using insights from studies in mammals and budding and fission yeasts. Finally, we discuss the differences in telomere homeostasis between normal cells and cancer cells, which may provide a foundation for telomere/telomerase targeted cancer treatments.

## 1. Introduction: chromosome maintenance and cell proliferation

### 1.1. Telomere homeostasis in normal and cancer cells

All dividing eukaryotic cells require telomeres to maintain the ends of the chromosomes and sustain chromosome stability. To protect the genetic information contained within the chromosomes, telomeres sacrifice their non-coding DNA sequences in the erosion that occurs during DNA replication in each cell cycle [1,2]. Most somatic cells that have undergone sufficient cell divisions to cause critical telomere shortening enter into replicative senescence. However, some cells, including lymphocytes, germ cells, stem cells and unicellular eukaryotes like yeast, express the enzyme telomerase, which has the ability to replenish telomeres and allow further replicative potential [3–7].

Telomerase is a ribonucleoprotein complex, composed of a reverse transcriptase enzyme catalytic subunit and a long non-coding RNA that contains the template sequence for telomere synthesis [6]. Whereas expression of the telomerase components is tightly regulated in differentiated cells, the vast majority of human cancers express active telomerase, effectively rendering them immortal [8–10]. A direct correlation between telomere maintenance and indefinite cell division was demonstrated *in vitro* by ectopic expression of telomerase in somatic cell culture [11]. However, while cancer cells stably maintain telomeres, they tend to be short [12,13]. In particular, some of them are critically short, termed ‘t-stumps’ [14], resulting in immortal cells that sustain a high risk of chromosome instability. This is strikingly different from our understanding of telomerase action in normal cells, in which telomerase preferentially elongates shorter telomeres until they are no longer short [15–17]. Why telomerase acts differently in cancer cells remains a mystery. In this review, we summarize our current knowledge of fundamental telomerase action, and highlight the phenotypes uniquely observed in cancer cells.

### 1.2. Proliferation and protection: the problems faced by telomeres

Progressive telomere shortening occurs each time a cell divides owing to incomplete replication of linear chromosome ends by the conventional DNA polymerases. This shortening is termed the end replication problem [18]. Nucleases also trim the telomeres to shape the chromosome ends for protection, thereby causing loss of telomeric DNA after S phase [19]. DNA replication-associated telomere shortening limits the number of divisions a cell can undergo, known as the Hayflick limit, before triggering the cessation of growth [20]. Once telomeres become critically short, the DNA damage response machinery is activated, and cells enter replicative senescence or undergo programmed cell death [21,22]. Telomere shortening leading to programmed cell death is a major tumour suppressor mechanism, and as such, most cancer cells require telomerase to be active in order to survive.

In the absence of the senescence checkpoint *per se*, critically short telomeres become ‘uncapped’; they lose their end protection ability. One essential role of the telomeres is the differentiation of bona fide chromosomal ends from damaged DNA double-stranded breaks [23,24]. This function is indispensable for maintaining chromosome integrity, as illicit repair of chromosome ends could result in chromosome fusions. Such fusions would induce mitotic arrest and cell death [25] or cause breakage–fusion–bridge cycles in subsequent cell divisions, leading to translocations, aneuploidy and eventually genomic instability [26]. This is called the end protection problem.

## 2. The structure of telomeres and telomerase

### 2.1. The shelterin complex and telomere conformation

Telomeres are specialized DNA–protein complexes found at the ends of all linear chromosomes. Telomeric DNA is composed of arrays of short guanine-rich tandem repeats, and while most of the telomere is double-stranded (ds), they terminate in a single-stranded (ss) G-rich 3′ overhang called the G-tail [24,27–29]. These telomeric ds and ssDNA repeats are covered by a specialized protein complex, termed shelterin, to evade recognition of the chromosome ends by the DNA damage response machinery. Together with the shelterin complex, telomeres establish a heterochromatin structure that packages up the ends of the chromosomes and prevents them from being aberrantly recognized as DNA double-stranded breaks (DSBs) [30]. The G-tail is thought not to be exposed, but rather hidden within the dsDNA by forming a displacement loop (D-loop). Telomeres are further compacted into a lasso-like structure called a t-loop with the aid of the proteins in the shelterin complex [31,32]. In addition, the shelterin complex interacts with DNA damage response factors, preventing induction of their downstream pathways. Thus, failure of the chromosome ends to interact with shelterin can expose the DNA ends and elicit the DNA damage response [23,24,33].

**Figure 1.**
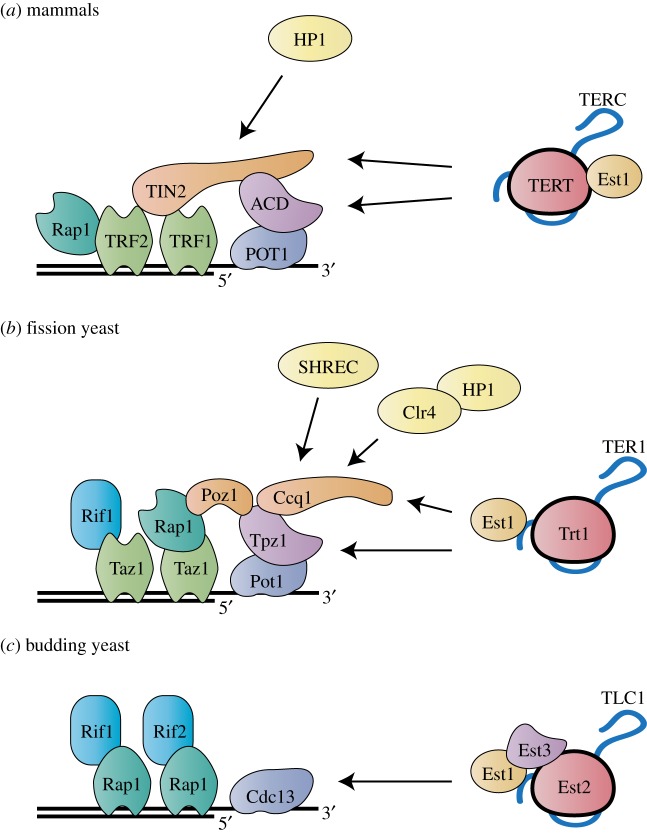
Shelterin conservation at the telomere from yeast to mammals. Schematic diagrams depict the proteins of the shelterin complexes and telomerase complex in (*a*) mammalian cells and (*b*) fission and (*c*) budding yeasts. Orthologous proteins are shaded in the same colour. Known interactions with the telomerase complexes and chromatin modifying proteins are indicated. Other interaction proteins are omitted from these diagrams.

In mammals, shelterin is composed of six proteins: TRF1, TRF2, RAP1, TIN2, ACD (previously known as TPP1) and POT1 (figure 1*a*) [34]. TRF1 and TRF2 bind the telomeric dsDNA and recruit TIN2, which associates with ACD. POT1 forms a heterodimer with ACD and directly binds to the telomeric ssDNA at the D-loop and the G-tail. Thus, the shelterin complex formation bridges the telomeric ds and ssDNA and stabilizes the telomeric proteins. It is also thought to negatively regulate telomere lengthening [35–40]. TRF2 is required for the formation of t-loops [31,32] and for suppressing ATM activation and non-homologous end-joining (NHEJ) of chromosome ends [41]. These functions are promoted by TRF2-dependent topological changes [42]. RAP1, which binds to TRF2, is also thought to play a role in inhibiting NHEJ and homology directed repair but its actual function remains debatable [43,44]. TRF2 and RAP1 may be redundantly required for telomere protection even though RAP1 localization is dependent on TRF2. Interestingly, TRF2 and RAP1 also bind to internal telomere sequences and modulate transcription [45]. TRF1 is dispensable for end protection, but is required for lagging strand synthesis during DNA replication [46]. POT1 blocks the binding of replication protein A (RPA) to the telomeric ssDNA, thereby preventing recruitment of ATR [23]. Among the members of the shelterin complex, TIN2 and ACD are responsible for recruiting telomerase to the telomeres (discussed in a later section).

The shelterin complex is well conserved between fission yeast and mammals (figure 1*b*) [34,47,48]. The telomeric dsDNA binding protein Taz1 (orthologue of TRF1 and TRF2 in mammals) supports the replication of telomeric DNA and inhibits the NHEJ pathway [49,50]. Pot1 directly binds the telomeric ssDNA to inhibit degradation of chromosome ends [51]. Like mammalian shelterin, Taz1 forms the shelterin bridging structure with Rap1, Poz1 (TIN2 orthologue), Tpz1 (ACD orthologue) and Pot1 [48]. Thus, fission yeast shelterin also connects the ds and ss telomeric DNA, and maintenance of this connection has been shown to negatively regulate telomerase activity [48,52–55]. Tpz1 also interacts with Ccq1, which recruits telomerase [48,56]. We believe that mammalian TIN2 may be a bifunctional protein orthologue of fission yeast Poz1 and Ccq1 (discussed later). While each protein has a distinct role, the overall formation of the shelterin complex is crucial for telomere maintenance and telomerase regulation.

Although a shelterin-like protein complex has not been found, budding yeast has been the best-studied model system for telomere biology. In this organism, Rap1 directly binds the telomeric dsDNA and, with its associated proteins Rif1 and Rif2, negatively controls telomerase action [57,58]. Cdc13 solely binds the telomeric ssDNA to recruit and stabilize telomerase [59–61]. Although these proteins do not associate to bridge the ds and ss telomeric DNA (figure 1*c*), each protein functions to maintain telomere structure and protect the chromosome ends. Importantly, the mechanisms and principles of telomeric protein-mediated telomere homeostasis observed in budding yeast appear to be largely conserved to fission yeast and mammals (conservation of the structure and function of telomeres and telomerase has been reviewed [24,47,62,63]).

### 2.2. Telomerase structure and accessory proteins in fission yeast and mammals

The core components of telomerase comprise the telomerase RNA (TER1 in fission yeast and TR or TERC in mammals) and the catalytic reverse transcriptase protein (Trt1 in fission yeast and TERT in mammals) [64]. The reverse transcriptase subunit has been well conserved throughout evolution [65]. It can be divided into three major structural and functional domains: a telomerase essential amino-terminal domain (TEN), a telomerase RNA-binding domain (TRBD) and a reverse transcriptase domain (reviewed in [66]). In contrast, the telomerase RNA varies widely in length and sequence between different organisms [67–69], and accommodates distinct RNA recognition proteins. However, some conserved functional elements exist, including the template domain, a template boundary element to limit the extent of reverse transcription, and a pseudo-knot domain important for binding to the telomerase catalytic protein [70]. While these two core components alone are required for *in vitro* telomere synthesis [71], the telomerase accessory proteins contribute to the assembly, stabilization and trafficking of telomerase (reviewed in [72]).

In fission yeast, the Sm family of proteins associate with the TER1 RNA, contributing to telomerase maturation and stability. Subsequent replacement of Sm with the Lsm2–8 complex promotes Trt1–TER1 interaction [73]. Est1 directly binds TER1 and directs telomerase to telomeres through an interaction between its 14-3-3-like domain and the shelterin component Ccq1 [74,75].

In mammals, telomerase RNA maturation uses ribosomal RNA biogenesis (reviewed in [76]). The telomerase RNA, TERC, is part of a group of RNAs called H/ACA and binds to a tetrameric complex, composed of the dyskerin, NAF1, NHP2 and NOP10 proteins [77,78]. This H/ACA ribonucleoprotein complex stabilizes TERC and ensures the localization of telomerase to small sub-nuclear organelles called Cajal bodies where NAF1 is replaced by GAR1 [79–81]. Once inside the Cajal bodies, TERC associates with TERT to form the mature telomerase complex. After the assembly of a minimal telomerase complex containing TERT, TR and dyskerin, interaction with a protein called TCAB1 facilitates trafficking of telomerase to the telomeres [82].

Mammalian telomerase biogenesis additionally requires the molecular chaperones heat shock protein 90 (HSP90) and P23, which bind TERT for assembly with TERC. They are also thought to provide a binding site for proteins which link to the dynein–dynactin motor, thereby promoting the transport of hTERT to the nucleus along microtubules [83]. In mammals, the yeast Est1 orthologue, EST1A (SMG6), interacts with TERT and can bind to the telomeric ssDNA [84]. However, Est1A is not directly involved in telomerase recruitment but rather telomere protection and maintenance [85]. It also plays a role in nonsense mediated-mRNA decay and appears to affect the abundance of telomeric RNA transcripts called TERRA [86], which contribute to the regulation of telomere length homeostasis (reviewed in [87]).

## 3. Fundamental mechanisms of telomerase action in yeasts and mammals

### 3.1. Telomerase expression and cellular proliferation

The level of functional telomerase enzyme expressed in a wide range of different cell types has been characterized using the telomeric repeat amplification protocol assay. This method essentially allows a measure of the telomerase activity contained within a cell lysate *in vitro* [9]. Using this assay, it has been well documented that most differentiated somatic cells lack detectable telomerase activity [9,10], explaining the propensity for telomere shortening through successive cell divisions [11–13,88].

Telomerase is, however, highly expressed in adult testes and ovaries, allowing consistently longer telomeres to be inherited by the next generation [4,13]. Telomerase remains active during early embryonic development but expression declines after the blastocyst stage and can no longer be detected in neonatal somatic cells [4,89,90]. Nevertheless, most stem cell populations possess weak telomerase activity [3,5,9,10], which is not sufficient to immortalize cells but does extend the proliferative ability of these self-renewal tissues (reviewed in [91,92]). Notably, the Hayflick limit of somatic cells can be indefinitely evaded when telomere length is maintained by high ectopic expression of telomerase [11]. Therefore, the level of telomerase expression defines telomere length homeostasis and proliferative capacity.

### 3.2. Common mechanisms for telomerase recruitment

**Figure 2.**
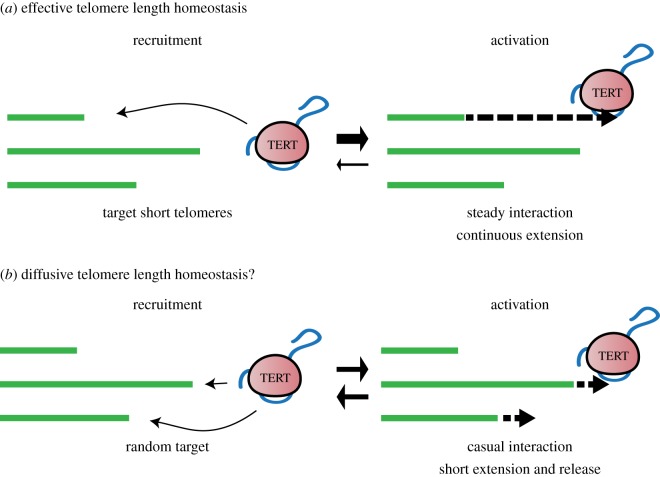
Models of telomere maintenance in normal and cancer cells. Schematic diagrams depicting two mode of telomere length homeostasis by distinct telomerase action. In normal cells (*a*), telomerase is recruited to the shortest telomere and it is extended until it is no longer short. Theoretically, such action would require a steady interaction between the telomere and telomerase, resulting in continuous extension. In this mode, distribution of telomere length would be the Gaussian like. In contrast, in cancer cells (*b*), telomerase recruitment appears to be random as it is independent of telomere length. Furthermore, telomeres are extended only a small amount, perhaps indicating an inability to maintain a steady interaction with telomerase, resulting in short extension and release. Such casual interaction permits repetitive recruitment of telomerase to new telomeres. In this mode, distribution of telomere length would be dispersal, resulting in retention of some short telomeres.

To maintain telomere length homeostasis, active telomerase needs to be efficiently recruited to every short telomere. Although telomeric proteins and telomerase components differ between budding and fission yeasts and mammals, on-going studies reveal that fundamental common features operate in telomerase action. In yeasts, telomerase is specifically recruited to shortened telomeres and the shorter telomeres are elongated the most during S phase [17]. As shorter telomeres can accommodate fewer telomeric DNA binding proteins, a quantitative negative regulation effect is thought to define the frequency of telomerase recruitment [93,94]. This system allows telomeric DNA to be retained at every chromosome end despite the presence of only a few molecules of active telomerase (figure 2*a*). This model has also been indirectly demonstrated in mammals using TERT heterozygous mouse cells [15].

The mechanism of telomerase recruitment was originally best defined in budding yeasts. Recruitment of telomerase during S phase is mediated by association of Est1 and the single-stranded telomeric DNA binding protein Cdc13 [95–99]. This interaction is triggered via phosphorylation of Cdc13 by the DNA damage response kinases Tel1/Mec1 (ATM/ATR orthologues) and the cell cycle coordinator Cdk1 [61,99–103]. As mentioned, telomerase recruitment occurs preferentially at short telomeres owing to a quantitative negative regulation effect of telomere-bound Rap1 [93,94].

Several studies have shown that recruitment of telomerase to the telomere in fission yeast occurs via direct interaction of the telomerase accessory protein Est1 with Ccq1, the telomeric Tpz1 (ACD orthologue) binding protein. This occurs after phosphorylation of Ccq1 by the Rad3 and Tel1 kinases (ATR and ATM orthologues respectively) [68,69,75,104,105]. However, recent work indicates that the Ccq1–Est1 interaction is likely to be transient, as association of Est1 with TER1 and Ccq1 is mutually exclusive [75,106]. Further stable association of telomerase at the telomere is achieved via interactions between Tpz1, Ccq1 and Trt1 [106,107]. The telomeric dsDNA binding protein Taz1 (TRF1/2 orthologue) restricts Rad3/Tel1 activation and telomerase recruitment at the telomere, limiting telomerase recruitment to S phase of the cell cycle [108,109]. Similar to the quantitative negative regulation effect observed in budding yeast, Taz1 suppresses activation of Rad3/Tel1 in a telomere length-dependent manner. Therefore, Rad3 and telomerase are efficiently recruited to short telomeres.

Mammalian telomerase recruitment also occurs in S phase of the cell cycle. The rest of the time it appears to be concentrated in Cajal bodies or elsewhere in the nucleus [110,111]. The POT1–ACD complex is known to associate with telomerase [112–114]. TIN2, the shelterin protein that bridges TRF1/2 and ACD, is crucial for ACD localization at telomeres and is also involved in telomerase recruitment [30,114–116]. Similar to yeasts, phosphorylation of a telomeric protein, ACD, occurs during S phase by a kinase, Cdk1, which is thought to increase the stability of its interaction with TERT [117]. However, this phosphorylation-mediated interaction remains debatable [118]. Therefore, how the interaction of ACD and telomerase is achieved remains to be elucidated. It is possible that TIN2 may function like Ccq1 in fission yeast and initiate the interaction between ACD and TERT. In support of this hypothesis, the *TIN2-R282H* mutation, which is found in patients with dyskeratosis congenita, impairs telomerase recruitment, whereas ACD recruitment and shelterin formation are unaffected [116,119]. Another similarity to the yeast recruitment process is the contribution of ATM/ATR signalling to telomere elongation. Phosphorylation of the shelterin component TRF1 at an ATM/ATR target site (S367) has been shown to increase telomerase recruitment [120,121]. As with yeasts, mammalian telomeres are also thought to have a quantitative negative regulatory effect, with proteins such as TRF1 and TRF2 negatively regulating telomere extension by telomerase [122,123]. However, quantification of TRF1 and TRF2 molecules on the telomeres suggests the number of TRF proteins is limited and long telomeres may not possess more [124,125]. Indeed, an alternative telomeric DNA binding protein, TZAP, binds to long telomeres in a manner that is mutually exclusive to TRF protein binding [126]. TZAP functions to trim the telomeres by excising the t-loop, thereby making long telomeres short. As TZAP counteracts the possession of very long telomeres, we anticipate that it may also function in restricting telomerase activity.

The three model systems described above highlight common fundamental features of telomerase recruitment. The DNA damage checkpoints appear to monitor and flag the shorter telomeres that harbour fewer numbers of the telomere dsDNA binding proteins. In fission yeast and mammals, a direct interaction between the OB (oligosaccharide/oligonucleotide)-fold domain of Tpz1 and ACD, especially the so-called TEL patch on the surface [127], and the TEN domain of the telomerase catalytic subunit is necessary for both telomerase retention and processivity [106,107,114,128–130]. Notably, mammalian TIN2 and fission yeast Ccq1, which bind to the C-terminus of ACD and Tpz1 respectively, are also required for telomerase recruitment, as demonstrated by loss of function mutations [104,116]. TIN2 and Ccq1 also recruit and associate with the heterochromatin proteins to control the status of condensation or cohesion at telomeres [131–135]. Owing to the similarities in function between TIN2 and Ccq1 in terms of end protection, telomerase recruitment and interaction with chromatin modifying proteins, it is tempting to speculate that Ccq1 may be the functional equivalent of mammalian TIN2. The connection between telomere architecture and telomerase accessibility is slowly being uncovered [30]. Both fission yeast and mammalian shelterin formation controls the extendible, non-extendible and extending states [52]. Thus, although the structural similarities of fission yeast and mammalian shelterin proteins are not great, their functions and roles are highly conserved.

### 3.3. Current models of telomerase activation

Once telomerase is at the telomere, the single-stranded 3′ overhang at the distal end of the telomeric DNA forms the substrate for telomerase. This anneals with the template region on the telomerase RNA to form a DNA/RNA hybrid, and one repeat of telomeric DNA can be added to the end of the 3′ tail using the complementary RNA sequence as a template. Telomerase then repositions its RNA template on the 3′ end of the substrate and adds another telomeric repeat. The ability of a single telomerase complex to add multiple repeats in a single cell cycle without dissociation is termed repeat addition processivity (RAP) [136,137]. The exact mechanism by which telomerase can reposition itself on the template remains to be elucidated. Single molecule imaging of telomerase revealed that rearrangement of the telomerase RNA molecule is coupled with catalytic action, posing a possible model for resolution of the DNA/RNA hybrid and translocation of the RNA template after synthesis [138]. Thus, it is becoming increasingly clear that multiple factors affect the processivity or activity (how fast nucleotides are added) of telomerase after it has been recruited to the telomere.

In mammals, stable association of telomerase with the POT1–ACD complex occurs after recruitment to telomeres. Binding of POT1–ACD to the telomeric ssDNA has been shown to decrease the rate of RNA primer dissociation, aid template translocation and enhance telomerase processivity *in vitro* [112,139]. Indeed, mutations within the TEL patch in the OB fold of ACD, which impair association with TERT, have been shown to decrease processivity of telomerase by POT1–ACD *in vitro*, compared with wild-type ACD [127,140]. Interestingly, however, another recent study found that the POT1–ACD complex increases not only telomerase processivity but also activity, resulting in more rapid dissociation from the primer [141]. Thus, ACD-mediated retention of TERT improves telomerase processivity and activity.

The TEN domain of mammalian TERT has dual functions. In addition to binding the OB-fold domain of ACD for telomerase recruitment and RAP stimulation, the TEN domain supports the stable formation of the RNA–DNA duplex in the active site of the enzyme [142–144]. The reverse transcriptase and C-terminal domains of telomerase have also been proposed to interact with the telomeric DNA substrate to help promote RAP [145,146]. Thus, while the TEN domain of TERT associates with ACD for recruitment, it also promotes stable association of TERT with the telomeric ssDNA for repeat synthesis. How these two activities of the TEN domain are coordinated remains to be elucidated.

Recent studies have begun to demonstrate that recruitment of telomerase to the telomere in S/G2 phase does not equate to activation of the enzyme. In mammals, a residue within ACD (L104), found on the opposite face of the OB fold to the TEL patch, has been implicated in regulating telomerase activity. Mutation of this residue causes short telomeres despite the mutant protein binding a similar amount of telomerase to mutant ACD proteins in cells with longer telomeres [118,127]. Intriguingly, a mutant form of the ACD orthologue in fission yeast, Tpz1 (K75A), also cannot maintain telomere length despite being fully capable of recruiting telomerase to telomeres [106,107]. Nevertheless, a stable interaction between Tpz1 and Trt1 is required for the processive activity of telomerase, as the Tpz1 (K75A) mutation could be overcome by fusion of Tpz1 directly to Trt1 [106]. However, telomerase activity requires Ccq1 association with the telomerase-bound Tpz1. It has been proposed that recruitment of telomerase by Ccq1 might temporally and locally resolve shelterin formation to allow access to the telomeric 3′ end [52]. Thus, these studies demonstrate that association of telomerase with telomeres is not sufficient to regulate telomere length and a subsequent activation step must exist.

In budding yeast, another telomerase component, Est3, complexes with Est1 and Est2 at the telomere in S/G2 phase to activate telomerase [96,97,147,148] (figure 1*c*). Est3 is composed of the OB-fold domain and interacts with the TEN domain of Est2 with the aid of Est1 [149]. It has been proposed that the surface residues on Est3 required for telomerase activation might be comparable to residue L104 in ACD [150]. Thus, in all three model organisms, telomerase activation requires a stable association with the OB-fold ACD family of proteins as well as conformational changes in telomere structure to provide telomerase with access to the ssDNA end.

### 3.4. Termination of telomerase activity

To understand the processivity of telomerase, we also need to know how telomerase action is terminated. Several factors at the telomeres can inhibit, rather than stimulate, telomerase processivity. The CST (CTC1, STN1 and TEN1) complex, of which homologues for STN1 and TEN1 exist in fission and budding yeast, is thought to terminate telomere elongation by recruiting DNA polymerase alpha to the ssDNA overhang, thereby displacing telomerase [151–153]. Studies in budding yeast have shown that Stn1 replaces Est1 as the Cdc13 binding partner and blocks further telomerase recruitment after S phase [154,155]. However, because telomerase can repeatedly access the same telomere for further extension during S phase, we may need to separately consider termination of processivity and inhibition of telomerase recruitment.

The lack of coupling between telomere extension and telomere lagging strand synthesis may itself lead to inhibition or termination of telomerase processivity. The presence of a long G-tail can encourage the formation of certain structural conformations in the telomeric DNA, such as G-quadruplexes, which could potentially inhibit the access of telomerase to the telomere [32,52,156]. The POT1–ACD complex plays a role in preventing the formation of such secondary structures [112,139], as does RPA in fission yeast [157]. As such, the extent of telomere extension may well be monitored/controlled by the amount of ssDNA binding proteins recruited to the telomere. Further investigations will be needed to define these differences and to reveal how telomerase is temporally released from the 3′ telomeric overhang to terminate processivity.

## 4. Telomere biology in cancer

### 4.1. Unique features of telomerase action in cancer

Telomerase reactivation or upregulation is a critical feature in the vast majority of cancers. While the mechanisms controlling hTERT expression are not fully understood, they are thought to include hTERT promoter mutations, alterations in alternative splicing of hTERT pre-mRNA, hTERT gene amplification, epigenetic changes and disruption of the telomere position effect machinery (reviewed in [158]). Many studies carried out in human cells in actual fact use established cancer cell lines, because primary cells senesce in cell culture without the induction of telomerase expression. The characterization of telomeres in cancer cells has revealed that activated telomerase can largely maintain telomere length homeostasis as well as cell proliferation. The average length at which telomeres are maintained directly correlates with the expression level of telomerase [159–161]. Nevertheless, despite the fact that many cancer cells express highly active telomerase [9,10], their telomeres are shorter than in paired differentiated normal tissue [162,163]. Strikingly, a subset of telomeres are left very short (t-stumps) in cancer cells [14]. Thus, although activated telomerase maintains chromosome ends overall, the manner in which the telomeres are maintained appears to differ from that in normal tissue, such as germ cells.

The reason for the persistent presence of short telomeres in cancer cells might originate from some modified action of telomerase. Our understanding of telomerase from studies in yeasts and murine embryonic stem cells is that it preferentially elongates shorter telomeres until they are no longer short [15,17]. In contrast, in cancer cells, the majority of telomeres are elongated, but they are only extended a short length [164]. A recent single cell live imaging study using HeLa cells demonstrated that human telomerase forms short dynamic interactions with the majority of telomeres, probing each chromosome end multiple times during S phase [165]. Thus, we predict that cancer telomerase targets every telomere but only extends them a little (figure 2*b*). This model would explain how some telomeres can be left at a short length or lost but the overall mean telomere length reflects/correlates with the amount of active telomerase.

Curiously, telomere extension by telomerase is not coupled with synthesis of the complementary C-rich strand by polymerase alpha [164], leading to long G-tail extensions during S phase. It has been proposed that telomerase recruitment/retention (and hence processivity) is terminated by recruitment of the CST complex, which associates with the polymerase alpha complex [151,152,166]. However, the probing interactions described in HeLa cells are only rarely converted into static interactions long enough to allow telomere elongation [165]. Thus, it is possible that telomerase might associate with telomeres in an unstable manner in cancer cells, and therefore it exhibits low processivity and dissociates before C-strand synthesis.

The hallmark of irregular telomerase action in cancer cells might also/otherwise stem from the presence of alternatively spliced hTERT mRNA isoforms. Alternative splicing events are commonly observed in the majority of cancer cells, and can both control transcript abundance and contribute to proteome diversity [158]. hTERT mRNA is alternatively spliced in a wide range of species [167], and a number of variants are co-expressed at significant levels in tumour and stem cells [168–170]. However, the regulation and function of these splice variants is not well understood. Expression of a major splice variant lacking most of the RT domain has been correlated with low telomerase activity in cancers [171,172], and a recent study has shown that the translated protein product can bind the telomerase RNA and suppress telomerase activity [173], presumably by competing with the fully functional hTERT isoform for TERC binding. Therefore, such deletions or substitutions of other key residues and domains may well affect the association of telomerase with shelterin components or the telomeric ssDNA overhang.

The shelterin complex proteins are important not only for telomerase recruitment but also for control of the DNA damage response and cell cycle control machineries at telomeres. These machineries are impaired or altered in cancer cells. Several mutations in genes encoding the components of the shelterin complex have also been identified in cancers. These mutations might affect telomere status and telomerase accessibility or processivity. For example, in familial cases of chronic lymphocytic leukaemia (CLL) loss-of-function mutations in POT1, affecting either its interaction with the telomeric ssDNA or with ACD, were found to co-segregate with CLL [174]. Furthermore, in melanoma patients, elongated telomeres were associated with mutations that impair telomeric ssDNA binding and Pot1–ACD–TIN2 interactions [175–178]. Such mutations would impair the complete formation of the shelterin bridge between the ds and ss telomeric DNA, disrupting the telomerase non-extendible state. Finally, in patients with myeloproliferative neoplasms, significantly reduced telomere length has been associated with elevated levels of POT1 and TIN2 expression, and downregulation of ACD and RAP1 compared with healthy controls [179]. It is not clear whether these alterations in the genes encoding the shelterin components are a cause or a consequence of the cancers. However, abnormal expression and/or function of the shelterin proteins is likely to contribute to telomere dysfunction, thereby driving genetic aberrations and cancer pathogenesis. At present, the factors contributing to the hallmarks of telomeres unique to cancer remain a mystery. However, growing data indicate that cancer cells may harbour impairments affecting the later stages of telomerase activity regulation, such as the targeting and processivity of telomerase, thereby leaving some telomeres short.

On top of aberrant telomerase action, overall shortening of telomeres could be consequences of selection. Random addition of telomeric repeats should lead to some very long telomeres. As the amount of telomeric proteins are limited, insufficient binding of the TRF proteins can cause fragile telomeres and constitutive DNA damage [46]. Hence, cells containing long telomeres are sensitive to further DNA damage and could be selectively eliminated by cell death [180]. Alternatively, homologous recombination or TZAP may become highly active in cancer cells, thereby actively trimming long telomeres. Further investigation into the functions of the telomeric proteins and their potential aberrations in cancer would benefit our understanding of how telomere homeostasis is differently maintained in cancer cells.

### 4.2. Targeting cancer telomeres and telomerase

Telomerase is an attractive potential drug target in the fight against cancer owing to its low/absent expression levels in normal somatic cells and high expression in cancer. Robust hTERT inhibition can lead to progressive telomere shortening and eventually cancer cell death. Thus, it should be possible to target cancer cells reasonably selectively, while the effect on normal cells should be minimal [181]. Several different compounds that directly target telomerase activity are currently under development, for example antisense oligonucleotides such as imetelstat/GRN163L (reviewed in [8]) and small molecules targeting hTR or hTERT such as BIBR1532 [182]. However, there is a long lag time between administration and clinical response for therapies that target telomerase activity, as telomeres must shorten before the effect is seen [8]. As such, therapies that target the non-canonical functions of telomerase, or which induce a DNA-damage response (DDR) at telomeres (i.e. 6-thio-dG, G-quadruplex stabilizers and oligonucleotides homologous to the 3′ telomeric overhang) may present a better treatment option [8,183].

Overexpression of an hTERT variant, which lacks most of the RT domain, was found to confer cells with a protective advantage against cisplatin-induced apoptosis, indicating a telomere homeostasis independent role for hTERT in cancer pathophysiology [173]. Thus, elucidating the connection between telomerase regulation and the regulation of RNA splicing has a great deal of potential to provide new insights into cancer biology [8]. Similarly, inhibition of the functions of the shelterin proteins could present a viable therapeutic option. For example, TRF1 is overexpressed in many types of cancer and plays an important role in telomeric DNA replication. Loss of TRF1 leads to uncapping of telomeres regardless of telomere length, and has been shown to impair lung tumour growth in mouse models [184]. However, it is not clear what effects targeting the shelterin proteins would have on normal cells.

It has been demonstrated that hTERT is also involved in upregulation of tRNAs [185] and WNT/β-catenin signalling [186] by interacting with their promoter regions. This non-canonical function of TERT appears to be TERC and RT activity-independent. Such aberrant transcription owing to TERT reactivation contributes to carcinogenesis. Therefore, inhibitors against multifunctional tankyrase, which is involved in telomere homeostasis, mitotic spindle formation and WNT/β-catenin signalling, and HSP90, which is involved in signal transduction, intracellular transport and protein degradation, have been explored to selectively kill cancer cells [183]. Finally, a number of immunotherapies are being tested in clinical trials, which aim to sensitize the immune system to tumour cells expressing protein fragments or peptides of telomerase on their cell surface. These are among the most promising telomerase targeting therapeutics, with hTERT specific immune responses being seen in telomerase positive tumours, minimal effects in normal cells and no autoimmunity (reviewed in [8]). Thus, there is a lot of potential in anti-telomerase therapeutics for cancer treatment, and a greater understanding of the regulation of telomerase expression, functions and activity can only serve to further enlighten the search for safe and effective treatments.

## 5. Conclusions and future perspectives

Until fairly recently, the activity of telomerase was thought to be controlled by limiting access to the telomeres. However, the collective data illustrate that telomerase recruitment and activation are separate events. Such a two-step mechanism is likely to be conserved from yeast to humans. However, the structural biology and biochemistry underlying the process of telomerase activation remains largely unknown and presents an important area for future research. In many human cancer cells, telomerase is highly expressed and recruited indiscriminately to all telomeres. Nevertheless, processivity is low, resulting in the maintenance of short telomeres. As such, both the preferential targeting of short telomeres and the processivity/activity of telomerase may be altered in cancer cells. We believe that t-stumps and altered telomerase regulation, such unique feature of telomeres in cancer, would be an ideal target for cancer therapeutics. Further investigation of telomerase regulation and action would benefit our understanding of the differences in telomere homeostasis between cancer and normal cells, and hopefully lead to the development of effective and safe anti-cancer treatments.

## 6. Take home messages

— Telomere function and action of telomerase are largely conserved between yeasts and mammals.
— The structural biology and biochemistry underlying the process of telomerase activation remains largely unknown, but emerging studies indicate evolutionary conservation of the mechanisms of telomere homeostasis from yeast to mammals.
— Telomere homeostasis differs between normal cells and cancer cells.
— The maintenance of short telomeres in cancer cells is thought to predispose to genomic instability, and indicates that the targeting and processivity of telomerase may be impaired in cancer.
— Telomeres and telomerase are attractive targets for anti-cancer therapeutics owing to its uniqueness in cancer cells, allowing selective targeting of cancer cells whilst having minimal effects on normal tissue.
